# Prevalence of Dental Caries and Traumatic Dental Injuries among 6- to 12-year-old Children in Bhopal City, India

**DOI:** 10.5005/jp-journals-10005-1429

**Published:** 2017-06-01

**Authors:** Satish Maran, ND Shashikiran, Pratibha Ahirwar, Priyanka Maran, Pawan Raj Kannojiya, Babita Niranjan

**Affiliations:** 1 Assistant Professor, Department of Pedodontics and Preventive Dentistry, Rishiraj College of Dental Sciences, Bhopal, Madhya Pradesh, India; 2 Dean and Head, Department of Pediatric and Preventive Dentistry, Faculty of Dental Sciences, Karad, Maharashtra, India; 3 Senior Lecturer, Department of Pedodontics and Preventive Dentistry, Bhabha Dental College, Madhya Pradesh, India; 4 Senior Lecturer, Department of Pedodontics and Preventive Dentistry, Institute of Dental Education and Advanced Studies, Madhya Pradesh India; 5 Senior Lecturer, Department of Pedodontics and Preventive Dentistry, Rishiraj College of Dental Sciences, Bhopal, Madhya Pradesh, India; 6 Senior Lecturer, Department of Pedodontics and Preventive Dentistry, Rishiraj College of Dental Sciences, Bhopal, Madhya Pradesh, India

**Keywords:** Dental caries, Dental trauma, Prevalence.

## Abstract

**Introduction:**

Dental caries and trauma are the most common oral health problems for many decades. There is need for prevalence data to analyze the nature of the problems and to take necessary steps in improving public health.

**Aim and objectives:**

To assess the prevalence of dental caries and traumatic dental injuries among schoolchildren of age 6 to 12 years in Bhopal city.

**Settings and design:**

Cross-sectional study design was selected. Universal sampling method was followed in this study.

**Materials and methods:**

A total of 1,204 children were examined. The distribution of samples was done based on age, gender, residing area, and type of school.

**Statistical analysis:**

Data were collected and statistically evaluated under chi-square test and analysis of variance.

**Results:**

The overall caries experience (73.17%) was found to be higher than that of traumatic injury experience (20.9%). There was age-related correlation between age and decay, missing, and filled teeth score.

**Conclusion:**

Since most injuries occur at home or at school, educating the individual is the key that will have a great impact on the prognosis of traumatic injuries. Also good food habits need to be instilled in children from a tender age with the help of parents, which is the ultimate solution to fight caries.

**How to cite this article:**

Maran S, Shashikiran ND, Ahirwar P, Maran P, Kannojiya PR, Niranjan B. Prevalence of Dental Caries and Traumatic Dental Injuries among 6- to 12-year-old Children in Bhopal City, India. Int J Clin Pediatr Dent 2017; 10(2): 172-176.

## INTRODUCTION

Caries and traumatic dental injuries are one of the most prevalent dental conditions in children. Despite various scientific advances and the fact that caries is preventable, the disease continues to be a major public health problem. In developing countries, changing lifestyles and dietary patterns had markedly increased the caries incidence. Traumatic dental injuries may occur throughout the life. But they are a particularly common unresolved problem throughout the world among schoolchildren. Also the trend in traumatic dental injuries is not well documented as that of dental caries.^1,2^ Epidemiological studies from various countries indicate that there is considerable variation in the prevalence of traumatic dental injuries. Hindsight explains that injury which can be foresight has to be prevented.^2^ Literature reviews shows that the prevalence of dental caries is falling more rapidly than traumatic dental injuries. If this trend continues, traumatic dental injuries may become more prevalent than dental caries.^3^

Therefore, a study was carried out to know the prevalence of traumatic dental injuries of anterior teeth fracture and also to compare with dental caries prevalence in school-going children of Bhopal city.

## MATERIALS AND METHODS

A cross-sectional study was designed. School-going children aged 6 to 12 years of Bhopal were randomly selected by probability sampling techniques. Bhopal city was divided into 14 zones (Bhopal Municipal Corporation) out of which two schools from each zone were selected. Out of the two schools selected, one was government and other one was private school. The list of schools was taken from the District Education office in Bhopal. Informed consent was taken from the parents/guardians of the children participating in the study 1 week prior to the examination, permissions were obtained from the concerned school authority, and ethical clearance was obtained from the Institutional Review Board.

### Inclusion Criteria

- Children and school authority willing to participate in the study
- Individuals whose consent had been obtained
- Participant within the age of 6 to 12 years
- Should be inhabitants of Bhopal city

### Exclusion Criteria

- Individuals whose informed consent was not obtained
- Children above or below 6 to 12 years of age
- Mentally/physically handicapped children
- Children not willing to participate in the study

Depending on the physical conditions of the school, exact arrangements for examination were made. Subjects were examined on an upright chair in adequate natural daylight and using diagnostic instruments. Examination was undertaken by a single examiner to avoid interexam-iner variability. Recording was done by a trained person who assisted throughout the study. Chemical sterilization was used to sterilize the instruments. Caries was recorded as per World Health Organization (WHO) criteria (1997). The Community Periodontal Index of Treatment Needs (CPITN) probe should be used to confirm visual evidence of caries on the occlusal, buccal, and lingual surfaces. The CPITN probe has a 0.5 mm ball at the tip and markings at 3.5, 8.5, and 11.5 mm. It has color coding from 3.5 to 5.5 mm. All maxillary and mandibular anterior teeth from canine to canine were examined for traumatic injury. Traumatic injuries affecting the teeth are clinically recorded based on objective signs and classified according to WHO Classification of Dental Trauma 1995 without the use of radiographs and pulp vitality testing. Results were tabulated and statistically evaluated.

## RESULTS

The present study was done to evaluate the prevalence of dental caries and traumatic dental injuries and was conducted on a sample of 1,204 children 6 to 12 years of age in Bhopal city, Madhya Pradesh. Table 1 shows the various factors associated with dental caries evaluated in the study. The percentage of caries prevalence in rural area (91.52%) with respect to urban area (0.47%) was statistically significant (p-value ≤0.0001). No significant difference in caries prevalence was seen with respect to gender (p-value ≥0.005). A significantly higher percentage of caries prevalence was seen in children of age 8 to 9 years. A significant difference was present when government school (42.19%) and private school (57.80%) were compared. Table 2 shows the various factors associated with dental traumatic injuries in children aged 6 to 12 years. A statistically significant corelation was seen when age and gender are compared, with 8-year-old students (28.8%) and males (57.1%) having a higher percentage. A statistically nonsignificant (p ≥ 0.05) relation was seen when residing area and type of school were compared. Table 3 shows the mean decay, missing, filling teeth (DMFT) score in the study population. The mean value of DMFT score of government school (2.36 ± 1.64) was higher than that of private school (2.37 ± 1.59). The mean value of DMFT score in rural area (3.41 ± 1.72) was higher than urban area (2.26 ± 1.58). Considering gender factor, the mean DMFT score value in females (2.36 ± 1.73) was higher when compared with males (2.17 ± 1.51). Table 4 shows paired t-test was applied to show the corelation of various factors associated with dental traumatic injury. A statistically significant (p ≤ 0.0001) corelation was seen when gender and residing area were compared. Table 5 shows the mean DMFT score in different age groups. A highest DMFT score was seen in children with age group of 9 years (2.83 ± 1.46). Table 6 shows one-way analysis of variance test in the age group of 6 to 12 years. On comparison, a highly significant difference was found between age and DMFT score with a p-value ≤0.0001).

**Table 1:** **Table 1:** Association between various factors and caries

*Factors*													
				*Caries status*									
				*Yes (%) 73.17*		*No (%) 26.81*		*Total (%)*		*Chi-square*		*p-value*
Residing area		Urban		785 (65.19)		317 (26.32)		1102 (91.52)		24.9		<0.0001	
		Rural		96 (7.97)		6 (0.49)		102 (8.47)					
Gender		Male		499 (41.44)		198 (16.44)		697 (57.89)		2.106		0.147	
		Female		382 (31.72)		125 (10.38)		507 (42.10)					
Age		6		84 (6.97)		48 (3.98)		132		40.879		<0.0001	
		7		200 (16.61)		54 (4.48)		254					
		8		198 (17.442)		77 (6.39)		275					
		9		186 (15.441)		36 (2.99)		222					
		10		99 (8.223)		42 (3.48)		141					
		11		60 (4.98)		48 (3.98)		108					
		12		54 (4.48)		18 (1.49)		72					
Type of school		Private		462 (38.37)		234 (19.43)		696 (57.80)		38.782		<0.0001	
		Government		419 (34.80)		89 (7.39)		508 (42.19)					

## DISCUSSION

Out of 1,204 children examined in the age group of 6 to 12 years, 881 (73.17%) children were showing positive caries experience in our study, and 7-year-olds showed a highest prevalence of caries, i.e., 200 (16.61%) children out of 1,204 with mean DMFT 2.1364, and the lowest prevalence of caries was observed in the 12 years age group, i.e., 54 (4.48%) children out of 1,204 (mean DMFT 1.7500).

The figures of prevalence of caries in age group of 8 to 9 years were also very close to that in 7 years age group, 16.61 and 15.44% respectively. The prevalence of caries for 6-year-olds was 6.97% (84 children out of 1,204). The prevalence of caries in the 11 years age group was 4.48%. This was much lower than the prevalence observed by Bauba et al^4^ with 79.48% and by Damle and Patel^5^ with 79.8%.

The relation of age with caries prevalence in the primary dentition was highly significant (p-value <0.0001). The DMFT scores declined progressively as age increased. This may be attributed to the loss of primary teeth as age advances as a result of normal exfoliative process. Rao et al^6^ reported similar reductions in DMFT scores in their study.

**Table 2:** **Table 2:** Association between various factors and traumatic dental injuries

				*Factors*											
				*Ellis class I (%)*		*Ellis class II (%)*		*Ellis class III (%)*		*Total*		*Chi-square*		*p-value*	
Gender		Male		48 (33.4)		66 (45.8)		30 (20.8)		144		20.6		<0.0001	
		Female		66 (61.2)		36 (33.3)		6 (5.5)		108					
Residing area		Urban		102 (44.7)		90 (39.4)		36 (15.7)		228		4.52		0.105	
		Rural		12 (50)		12 (50)		0		24					
Type of school		Government		72 (44.4)		66 (40.7)		24 (14.8)		162		0.442		0.802	
		Private		42 (45.6)		36 (39.1)		12 (11.0%)		92					
Age		6		12 (100)		0		0		12		95.1		<0.0001	
		7		24 (33.3)		6 (16.6)		6 (16.6)		36					
		8		24 (33.3)		48 (66.6)		0		72 (28.8)					
		9		0		6 (33.3)		12 (66.6)		18					
		10		18 (40.9)		18 (40.9)		6 (13.6)		44					
		11		24 (50)		18 (37.5)		6 (12.5)		48					
		12		12 (50)		6 (25)		6 (25)		24					

**Table 3:** **Table 3:** The DMFT score in the sample population

*DMFT score*							
				*N*		*Mean ± Std. Deviation*	
Type of school		Government		696		2.3621 ± 1.64853	
		Private		508		2.3720 ± 1.59306	
Area of residence		Urban		1102		2.2695 ± 1.58127	
		Rural		102		3.4118 ± 1.72548	
Gender		Male		697		2.1722 ± 1.51494	
		Female		507		2.6331 ± 1.73077	

**Table 4:** **Table 4:** Association between various factors and DMFT score

*Factors*		*T*		*p-value*	
Gender		4.907		<0.0001	
Residing area		–6.924		<0.0001	
Type of school		–0.105		0.916	

**Table 5:** **Table 5:** Mean DMFT score in different age groups

*Age*		*N*		*Mean ± Std. Deviation*	
6		132		2.1364 ± 2.03696	
7		254		2.7598 ± 1.77861	
8		275		2.4436 ± 1.37479	
9		222		2.8378 ± 1.46476	
10		141		1.9574 ± 1.29213	
11		108		1.5000 ± 1.46931	
12		72		1.7500 ± 1.43154	
Total		1204		2.3663 ± 1.62469	

**Table 6:** **Table 6:** Correlation between age and DMFT score

		*Sum of squares*		*Mean square*		*p-value*	
Between groups		229.292		38.215		<0.0001	
Within groups		2946.179		2.461			
Total		3175.471					

Out of 1,204 children, 697 (57.89%) were males and 507 (42.10%) were females, which were low compared with studies by Bauba et al,^4^ where 79.48 and 79.8% prevalence was found in males and females respectively. Out of 697 (57.89%) males, 499 (41.44%) were showing positive caries experience, whereas out of 507 (42.10%) females, 382 (31.72%) were showing positive caries experience. The prevalence of dental caries in males in the age group of 6 to 12 years was higher (41.44%) as compared with female students (31.72%). This is in conformity with the studies by Shetty and Tandon^7^, and Gaikwad and Indurkar,^8^ who reported higher caries experience in boys than in girls. The difference could be attributed to different age groups and geographic locations surveyed.

Out of 1,204 children residing in urban area, 785 (65.19%) were showing positive caries experience, whereas caries experience in children residing in rural areas was 96 (7.97%). The prevalence of dental caries in children residing in urban area for the age group of 6 to 12 years was higher (65.19%) when compared with children residing in rural area (7.97%). The prevalence of dental caries in children studying in private schools for the age group of 6 to 12 years was higher (38.37%) when compared with children studying in government schools (34.80%). This can be attributed to either low socioeconomic status and oral hygiene maintenance, but the differences in the DMFT scores were not statistically significant (p-value = 0.916). On the contrary, Singh et al^9^ reported a negative association between socioeconomic status and caries prevalence and severity.

Similar to dental caries, the traumatic injuries were also related to age, sex, and area of residence. There has been an array of classification used in previous studies for the prevalence of traumatic injuries, whereas in our study Ellis classification for traumatic injuries was used, as it is a simplified classification used by various studies for recording dental trauma. Our study includes Ellis class I, II, III classifications as its evaluation requires minimum time with no investigation or exposure to radiation required. In the present study, parents were not involved as children in school who suffered from dental injuries were taken. In the present study, among 1,204 schoolchildren examined, 252 children had fractured teeth, giving the overall prevalence rate of 20.9%. This result was not higher to that found by Navabazam^10^ (27.5%) in Yazd, Iran, in 9- to 14-year-old schoolchildren. In our study, the prevalence of traumatic injuries is about 4.76% at the age of 6 years, reaching about 28.8% at the age of 8 years. In agreement with previous observations, children of 8 to 10 years were often injured. Prevalence of trauma increases with increasing age. This may be attributed to the increasing mobility and activity with age or could be explained by the fact that dental injury is a cumulative defect.^10^

The finding that boys sustained more dental injuries than girls in our study was in accordance with other studies from different parts of the world. In our study, the prevalence ratio between boys and girls was about 4:3. The relatively low prevalence of trauma among girls can be explained by the fact that girls are generally more mature in their behavior than boys. Moreover, boys tend to be more energetic and inclined toward vigorous outdoor activities.^11^ The restricted and mature behavior of females enforced by conservative parents due to cultural and religious reasons could be a possible factor, which contributes to the low prevalence of trauma among Indian girls. Thus, behavior can be a very important factor in the occurrence of traumatic dental injuries among children, so this factor must always be taken into consideration in developing effective preventive strategies for preserving dental health, especially in children with high physical activities. This is in contrast with a study conducted in the city of Santo Domingo, Dominican Republic, done by Garcia-Godoy et al^12^ where girls (50.6%) are more affected by traumatic dental injuries than boys (48.7%). Other studies done by Rocha and Cardoso^13^ also showed an increasing trend of traumatic dental injuries among girls, because of their increasing participation in sports or activities formerly practiced by boys only.

The present study included both urban and rural areas, partly to estimate an average of new dental injuries in the population, and partly to investigate whether differences existed. In accordance with the study done by Cortes et al,^14^ a lower incidence was observed in the rural area. The reason for this geographic difference may be due to less involvement in sports activities in rural school-going children. Similar results were reported by Skaare and Jacobsen.^15^ Higher prevalence in private school can be correlated with sports-related injuries in the child. Our finding corroborated with those reported in Kaba and Marechaux.^16^

## CONCLUSION

Traumatic dental injuries and dental caries constitute a major public health problem creating not only physical but also psychological effects in children and their parents. Unfortunately, the public is unaware of the risk and does not have enough information to prevent dental caries and to avoid traumatic injuries to the teeth. On the contrary, health professionals, including dentists, underestimate the incidence of dental caries and dental trauma and concentrate on the treatment rather than prevention.

## CLINICAL SIGNIFICANCE

It is well known that prevention is always better as well as the most desirable action compared with treatment. By knowing the prevalence rate of dental caries and traumatic dental injuries among schoolage children, we can educate children, parents, teachers, and health care professionals and physical education teachers for the emergency care after a traumatic injury has occurred and also make them aware of the importance of deciduous dentition and the ways of caries prevention.
